# Xenotransplantation of the Porcine Lung—From Immunological Barriers to Emerging Experimental Strategies

**DOI:** 10.1111/xen.70169

**Published:** 2026-09-17

**Authors:** Nathalie Roters, Niveditha Varma, Robert Ramm, Heiner Niemann, Patrick Zardo, Arjang Ruhparwar, Tobias Goecke

**Affiliations:** ^1^ Department for Cardiac, Thoracic, Transplantation and Vascular Surgery Hannover Medical School Hannover Germany; ^2^ Lower Saxony Center for Biomedical Engineering, Implant Research and Development Hannover Germany; ^3^ Biomedical Research in End Stage and Obstructive Lung Disease Hannover (BREATH) German Center for Lung Research (DZL) Hannover Germany; ^4^ Department of Gastroenterology, Hepatology, Infectious Diseases and Endocrinology Hannover Medical School Hannover Germany

**Keywords:** chronic rejection, complement activation, fibrosis, gene editing, immune rejection, ischemia‐reperfusion injury, lung transplantation, T cell‐mediated rejection, tolerance induction, xenotransplantation

## Abstract

Lung transplantation is the only long‐lasting life‐saving treatment for patients with end‐stage pulmonary disease, but the ever‐growing shortage of suitable donor organs leads to high mortality rates of the afflicted patients. Xenotransplantation, the transplantation of organs across species, has emerged as a promising alternative. This review summarizes the current knowledge on immunological barriers in lung xenotransplantation, including mechanisms of hyperacute, delayed, and chronic rejection. We highlight potential experimental strategies to overcome the various rejection responses. Xenotransplantation of organs such as hearts and kidneys has recently been advanced to clinical trials. However, lung xenotransplantation remains highly challenging due to its specific vascular sensitivity, extraordinary immunogenicity, and the immediate exposure to the external environment. Thus, translating these advances into durable clinical graft function will require lung‐specific genetic and pharmacological strategies that directly address the organ's unique immunological vulnerabilities, especially its susceptibility to complement‐mediated vascular injury, microvascular coagulopathy, and pulmonary intravascular macrophage activation.

## 1 Introduction

According to the Global Observatory on Donation and Transplantation, only 20%–25% of patients on organ waiting lists worldwide receive a suitable transplant each year, with the gap between listed patients and actual transplants growing steadily. For lung transplants in particular, data from the Organ Procurement and Transplantation Network/Scientific Registry of Transplant Recipients (OPTN/SRTR) and Eurotransplant report indicate that only 17%–24% of listed lung transplant candidates undergo transplantation each year, while up to 15%–20% die on the waiting list or become too ill to undergo any transplantation [1]. This devastating scenario has prompted intense research into xenotransplantation around the world.

The domestic pig has been identified as the best‐suited donor species due to a number of specific advantages. When transplanting porcine organs into primates, several immunological rejection responses are induced that would destroy the xenograft within minutes or few hours, including the hyperacute rejection response (HAR), the acute vascular rejection (AVR) and finally the cellular rejection [2, 3]. The primary goal is to control the HAR and the AVR by genetic modification of the donor pigs and an appropriate immune suppressive treatment of the recipient. This requires production of multi‐transgenic pigs and extensive testing of their suitability for successful xenotransplantation. Intensive research around the globe has demonstrated that both, the HAR and AVR can be reliably controlled via knockouts of specific antigenic surface epitopes (e.g., galactose‐α1,3‐galactose (α1,3‐Gal) and simultaneous transgenic expression of human genes with anti‐inflammatory, anti‐apoptotic and anti‐coagulative function [2, 4]. Following orthotopic porcine organ transplantation into non‐human primates (NHPs), maximum survival rates of 264 days (heart) and 758 days (kidney) could be achieved [5, 6]. In contrast, xenogeneic lung transplantation is still at a rather early stage of development, with a maximum graft survival of 31 days in NHPs [7]. Data regarding previous experiments in xeno‐lung transplantation are shown in Table 1.

**TABLE 1:** Summary of preclinical pig lung xenotransplantation studies.

Study	Model type	Survival & gas exchange	PVR / hemodynamics	Platelet & neutrophil dynamics	Complement & coagulation	Key modifications & therapy	Principal advance & residual gap
**Burdorf et al., 2022** *Am J Transplant*	Pig‐to‐baboon (NHP, in vivo).	GalTKO.hCD46 lungs failed rapidly (90–240 min). Addition of hEPCR, hTBM or hEPCR/hTFPI enabled transient life‐supporting graft function, although most recipients developed progressive PVR elevation and tracheobronchial flooding within 24 h. Combined hEPCR+hTBM with anti‐inflammatory therapy resulted in more sustained function, with three recipients surviving 7, 9 and 31 days	Progressive PVR elevation and loss of vascular barrier integrity were the defining early injury events across genotypes. Combined hEPCR+hTBM expression with A1AT improved hemodynamic stability beyond 24 h.	βTG release and IL‐6 levels were reduced in hTBM‐containing combinations. Standard anti‐adhesion regimens did not consistently attenuate platelet, neutrophil, or monocyte sequestration	Vascular barrier injury and coagulation dysregulation persisted across genotypes. Humoral immune activation occurred despite αCD40 blockade, suggesting a role for residual non‐Gal antigens.	GalTKO.hCD46 ± hEPCR, hTBM, hTFPI, hCD47, HO‐1, β4GalKD. Adjuncts: A1AT, αCD40 mAb, BIA, histamine blockade, ± macrophage depletion.	Demonstrated multi‐week survival in a primate lung xenotransplantation model (up to 31 days), providing proof‐of‐principle for combined genetic and pharmacologic targeting. However, vascular barrier failure remained a major limitation and the minimal effective modification strategy remains undefined.
**He et al., 2025** *Nat Med*	Pig‐to‐human (brain‐dead recipient).	Observation period was 9 days in a brain‐dead human recipient. Gas exchange was maintained, with PaO_2_ around 100 mmHg at FiO_2_ 40%, and lung compliance and oxygenation remained stable over time.	Serial monitoring of lung compliance and respiratory pressures indicated stable respiratory mechanics. Detailed pulmonary hemodynamic parameters such as pulmonary vascular resistance were not reported.	Platelet and neutrophil dynamics were not reported. Histology showed CD3+ T cell and CD68+ macrophage infiltration at POD 3, 6 and 9, without numerical quantification.	Complement activation was indicated by progressive C4d deposition. IgG and IgM deposition increased over time, accompanied by rising circulating anti‐donor antibodies.	+ hCD55, hCD46, hTBM. Immunosuppression: rATG, MMF, tacrolimus, basiliximab, rituximab, eculizumab, tofacitinib.	First evaluation of a multi‐gene‐edited pig lung in a brain‐dead human recipient demonstrating sustained gas exchange over a 9‐day observation period. However, progressive antibody‐mediated injury occurred, indicating that durable control of humoral immunity remained unresolved.
**Chaban et al., 2023** *Xenotransplantation*	Human‐blood EVLP; elective 4‐h endpoint.	GalTKO.hetero‐CD46 lungs showed a median perfusion survival of 152 min (range 5–240), with 6/23 grafts reaching 4 h. Increasing CD46 gene dose and addition of hCD55 progressively improved perfusion survival, with 6/6 GalTKO.homoCD46+hCD55 lungs maintained for 4 h. Gas exchange was not assessed.	Biphasic PVR kinetics were observed across all groups, with an early transient rise followed by a later gradual increase. Genotype and CD46 gene dose did not significantly alter PVR kinetics.	Circulating neutrophils rapidly declined during perfusion, consistent with pulmonary sequestration, particularly in GalTKO.homo‐CD46+hCD55 lungs. Platelet activation markers (CD62P and βTG) were reduced with increasing CD46 expression and addition of hCD55	C3a levels were similar across genotypes despite increased CD46 expression, whereas C4d deposition increased with stronger CD46 expression. Microvascular thrombosis frequency was reduced in homo‐CD46 and homo‐CD46+hCD55 lungs	GalTKO.hetero‐CD46, homoCD46, homoCD46+hCD55. No pharmacological agents.	Demonstrated a gene‐dose effect of hCD46, with homoCD46+hCD55 lungs achieving 100% 4‐h perfusion survival and reduced thrombotic injury. Complement activation (C3a) was not reduced by increased CD46 expression, and the significance of late C4d deposition remains unclear.
**Miura et al., 2022** *Xenotransplantation*	Human‐blood EVLP; elective 8‐h endpoint	GalTKO.hCD46.hTFPI/hCD47 lungs were maintained for the full 480‐min perfusion period (7/7), whereas only 3/5 reference GalTKO.hCD46 lungs reached 480 min. Pulmonary oedema was markedly reduced, reflected by a lower wet‐to‐dry ratio	Pulmonary vascular resistance remained relatively low and stable in GalTKO.hCD46.hTFPI/hCD47lungs. Selectin/integrin inhibition showed a non‐significant trend toward attenuation of the initial PVR rise	Neutrophil sequestration was delayed in GalTKO.hCD46.hTFPI/hCD47 lungs, with higher circulating neutrophil levels during early perfusion. Platelet activation markers (CD62P and βTG) were reduced, and additional selectin/integrin inhibition did not further decrease cellular sequestration	Coagulation activation was markedly reduced, reflected by lower F1+2 levels consistent with inhibition of the tissue‐factor pathway by TFPI. Complement markers were not assessed.	GalTKO.hCD46.hTFPI/hCD47, BIA, anti‐GPIb Fab, histamine blockade, desmopressin ± PSGL‐1, GMI‐1271, IB4.	Demonstrated that hTFPI/hCD47expression reduces coagulation activation, platelet activation and pulmonary oedema, and delays early neutrophil sequestration. However, early platelet sequestration persisted, indicating that additional adhesion pathways remain active.
**Connolly et al., 2021** *Xenotransplantation*	Human‐blood EVLP (8 h) + pig‐to‐baboon (in vivo).	Ex vivo perfusion survival exceeded 8 h in both h‐pvWF and reference lungs, with most experiments reaching elective termination. In vivo, h‐pvWF lungs supported recipient survival up to 7 days compared with 4 days in reference recipients.	Pulmonary vascular resistance remained low during ex vivo perfusion in both groups, indicating no adverse hemodynamic effect of h‐pvWF. In vivo, the increase in peak airway pressure was attenuated in h‐pvWF recipients.	Platelet sequestration was markedly reduced and delayed in h‐pvWF lungs, particularly early during perfusion. Platelet activation was attenuated, whereas βTG levels and neutrophil counts were unchanged.	Platelet sequestration was markedly reduced and delayed in h‐pvWF lungs, particularly early during perfusion. Platelet activation was attenuated, whereas βTG levels and neutrophil counts were unchanged	GalTKO.hCD46. h‐pvWF. BIA, anti‐GPIb Fab, histamine blockade ± GMI‐1271, PSGL‐1, IB4.	Humanization of pvWF mitigates the pvWF–GPIb platelet incompatibility, reducing early platelet sequestration in both ex vivo and in vivo models. However, no clear survival benefit was observed and residual platelet adhesion persisted.

Abbreviations: βTG, β‐thromboglobulin; BIA, 1‐benzylimidazole (thromboxane synthase inhibitor); EVLP, ex vivo lung perfusion; F1+2, prothrombin fragment 1+2; FiO_2_, fraction of inspired oxygen; GalTKO (GTKO), α1,3‐galactosyltransferase knockout; h*pvWF, humanized porcine von Willebrand factor; hCD46/hCD55/hTBM/hEPCR/hTFPI/hCD47, human transgenic complement and coagulation regulators; NHP, non‐human primate; PaO_2_, arterial partial pressure of oxygen; POD, postoperative day; PVR, pulmonary vascular resistance; WDR, wet‐to‐dry ratio.

## 2 Lung‐Specific Barriers to Xenotransplantation

While xenotransplantation of porcine hearts and kidneys has been advanced to first clinical application [8, 9, 10, 11], and even the porcine liver has recently moved beyond the ex vivo stage with a gene‐edited pig liver remaining functional for 10 days in a brain‐dead recipient [12, 13], the lung continues to lag far behind. As summarized in Table 1, survival after pig lung xenotransplantation in NHPs is still limited to a few weeks at most, the longest reported being 31 days in a pig‐to‐baboon model using donor lungs lacking the α‐1,3‐galactosyltransferase gene and expressing human CD46 (GalTKO/hCD46), together with additional genetic modifications and pharmacological treatment [7]. Even with genetically modified donor lungs and intensive immunosuppression, lung xenografts show significantly higher rejection rates than other organs in preclinical NHP models [7, 14]. This vulnerability is closely linked to persistent complement activation, pulmonary edema, and thrombotic microangiopathy (TMA). Notably, even perfusion of porcine lungs with human plasma alone, in the absence of any immune cells, is sufficient to induce endothelial damage, complement deposition (C5b‐9), and interstitial hemorrhage [7, 15].

### 2.1 Anatomical and Physiological Particularities of the Porcine Lung

Several features of the porcine lung are different from other donor organ and may require different treatments when used as functional xenograft.

Due to anatomical differences, in most experimental settings only the left pig lung has been used for transplantation. Similar to humans, the porcine left lung consists of two lobes that are connected to the trachea through a single bronchus. The right pig lung, by contrast, comprises four lobes (cranial, middle, accessory, and caudal), whereas the human right lung has only three lobes. A distinctive feature of the porcine right lung is that the cranial (upper) lobe is ventilated by a separate tracheal bronchus arising directly from the trachea, cranial to the right main bronchus, while the remaining lobes are supplied with air by bronchi branching from the main bronchial tree [16].The accessory lobe, makes transplantation of the right pig lung surgically extremely demanding and may contribute to poor outcomes when used [16].

Beyond these specific macroscopic anatomical features, respiratory mechanics and gas exchange also differ between porcine and primate lungs. Higher airway resistance and reduced compliance, owing to a different airway geometry and alveolar structure, may compromise ventilation and oxygenation and could affect long‐term graft function after transplantation into humans [17]. This concern is largely extrapolated from comparative respiratory physiology and has not been directly quantified in lung xenografts. Pigs are also susceptible to ventilation‐induced lung injury and are therefore frequently used as a model for studying ventilator‐induced lung injury [18], which is relevant because xenograft recipients typically require prolonged mechanical ventilation.

Isoform differences in surfactant proteins between porcine and primate lungs have also been described [19]. Such structural differences could alter the local immune milieu and predispose to alveolar dysfunction and collapse, although a direct causal contribution to xenograft failure remains hypothetical at present.

Unlike solid organs such as the heart or kidney, the lung is continuously exposed to environmental antigens, pathogens, and pollutants and therefore depends on a specialized local mucosal immune defense. This constant exposure may raises the baseline level of immune activity in the graft and complicates immunosuppression, which must control xenoreactivity without leaving the recipient defenseless against inhaled pathogens.

A further barrier without a direct human equivalent is the population of pulmonary intravascular macrophages (PIMs) that reside inside the porcine pulmonary capillaries. In addition to their role in pathogen clearance, PIMs can release thromboxane and pro‐inflammatory cytokines upon xenogeneic exposure and thereby contribute to early graft injury. Depletion of intravascular macrophages from pig lungs using liposomal clodronate has been shown to prevent acute rejection in perfusion models, underlining their functional relevance [20, 21]. The precise molecular crosstalk between porcine PIMs and recruited human monocytes is largely unknown at present, either direct cell‐to‐cell contact may amplify the inflammatory cascade or the paracrine cytokine signaling may contribute to the crosstalk.

**FIGURE 1:**
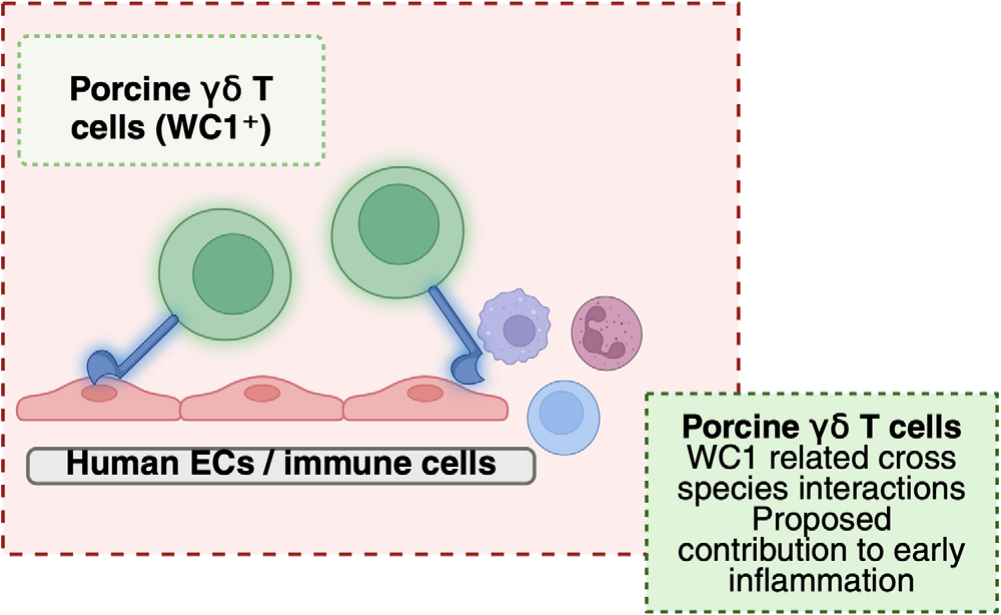
Proposed role of porcine γδ T cells (WC1^+^) in lung xenotransplantation. Porcine γδ T cells are far more abundant in pigs than in humans and express WC1 molecules that are absent in humans. The schematic drawing shows how these cells may engage human endothelial and immune cells through unconventional cross‐species interactions that could contribute to early immune activation and graft inflammation. As this role has not yet been demonstrated in lung xenotransplantation, all interactions are shown as proposed (dashed). Abbreviations: CD, cluster of differentiation; WC1, workshop cluster 1; γδ, gamma‐delta.

**FIGURE 2:**
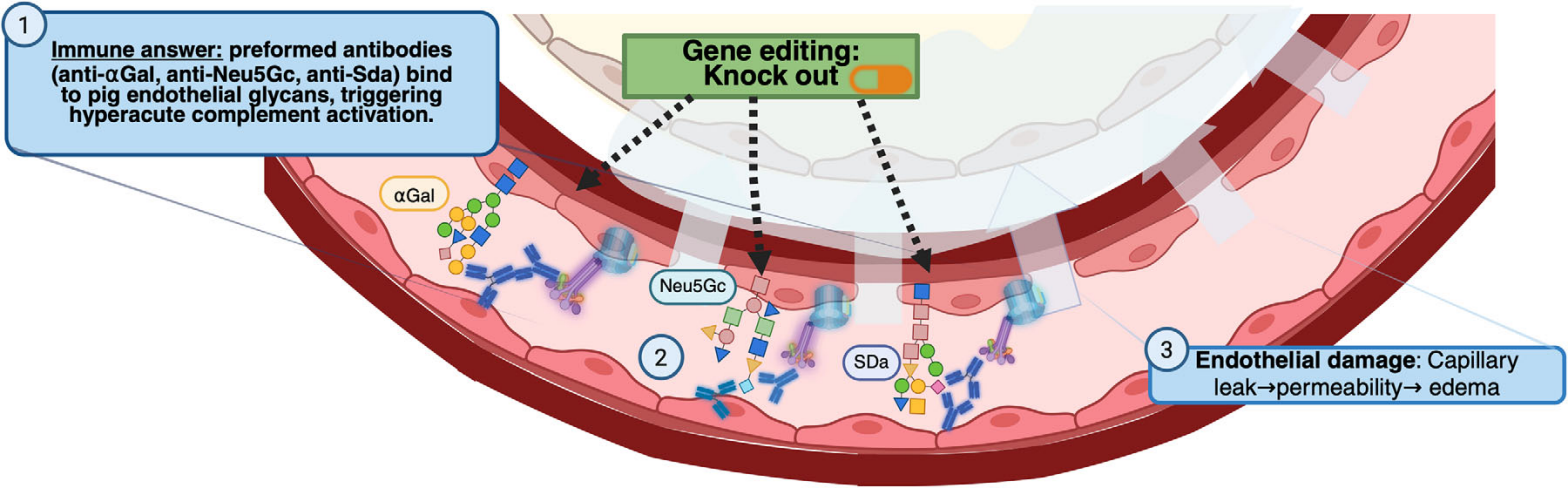
Immunological rejection cascade in porcine lung xenotransplantation. Sequential immune barriers, read from (1) binding of preformed xenoreactive antibodies (anti‐αGal, anti‐Neu5Gc, anti‐Sda) to porcine endothelial glycans, through (2) hyperacute complement activation, to (3) endothelial damage with capillary leak, increased permeability, and edema. Genetic knockout of the corresponding glycan antigens removes the initiating targets. Abbreviations: AMR, antibody‐mediated rejection; Neu5Gc, N‐glycolylneuraminic acid; Sda, Sid blood group antigen; αGal, galactose‐α‐1,3‐galactose.

**FIGURE 3:**
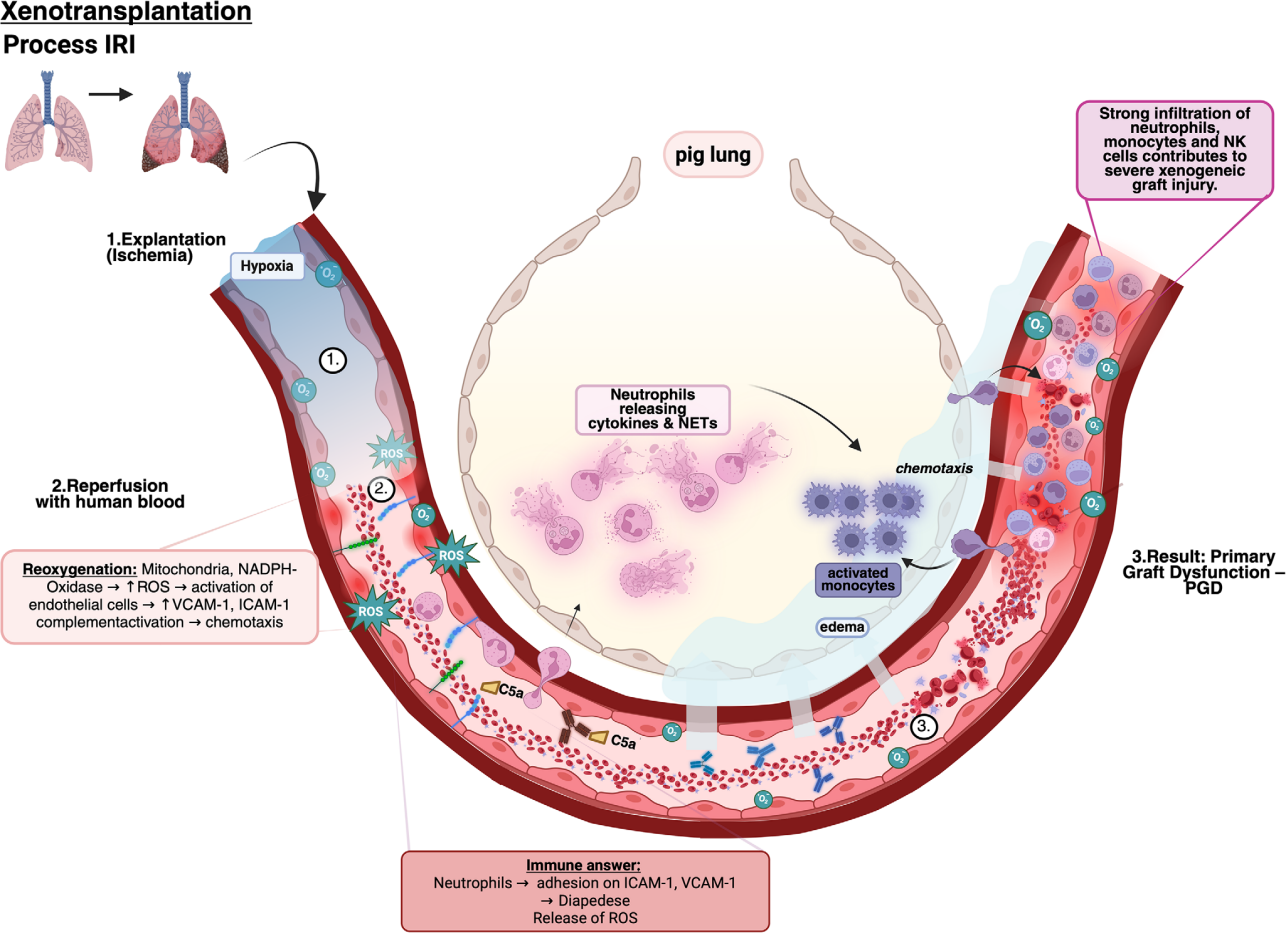
Ischemia‐reperfusion injury in porcine lung xenotransplantation. The three sequential stages are read as (1) explantation and cold ischemia with hypoxia and ATP depletion, (2) reperfusion with human blood, during which mitochondrial and NADPH‐oxidase‐derived ROS drive endothelial activation (ICAM‐1, VCAM‐1), neutrophil and monocyte adhesion and diapedesis, and complement activation, and (3) resulting in primary graft dysfunction with interstitial edema. Abbreviations: ATP, adenosine triphosphate; C5a, complement component 5a; ICAM‐1, intercellular adhesion molecule 1; NADPH, nicotinamide adenine dinucleotide phosphate; NET, neutrophil extracellular trap; PGD, primary graft dysfunction; ROS, reactive oxygen species; VCAM‐1, vascular cell adhesion molecule 1.

**FIGURE 4:**
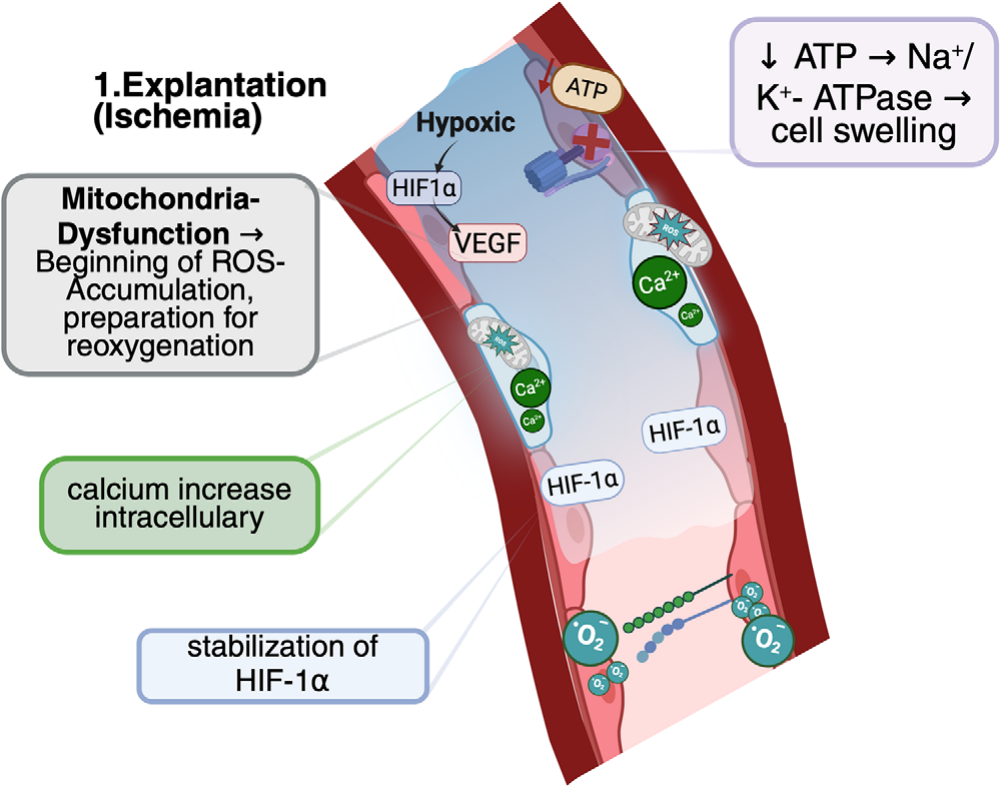
Cold ischemia and preservation injury during porcine lung explantation. Cellular and molecular events during warm ischemia at procurement and cold static storage: falling ATP impairs the Na+/K+‐ATPase and causes cell swelling, mitochondrial dysfunction begins ROS accumulation, intracellular calcium rises, and HIF‐1α is stabilized (with VEGF induction), together priming the endothelium for subsequent reperfusion injury. Abbreviations: ATP, adenosine triphosphate; HIF‐1α, hypoxia‐inducible factor 1‐alpha; ROS, reactive oxygen species; VEGF, vascular endothelial growth factor.

**FIGURE 5:**
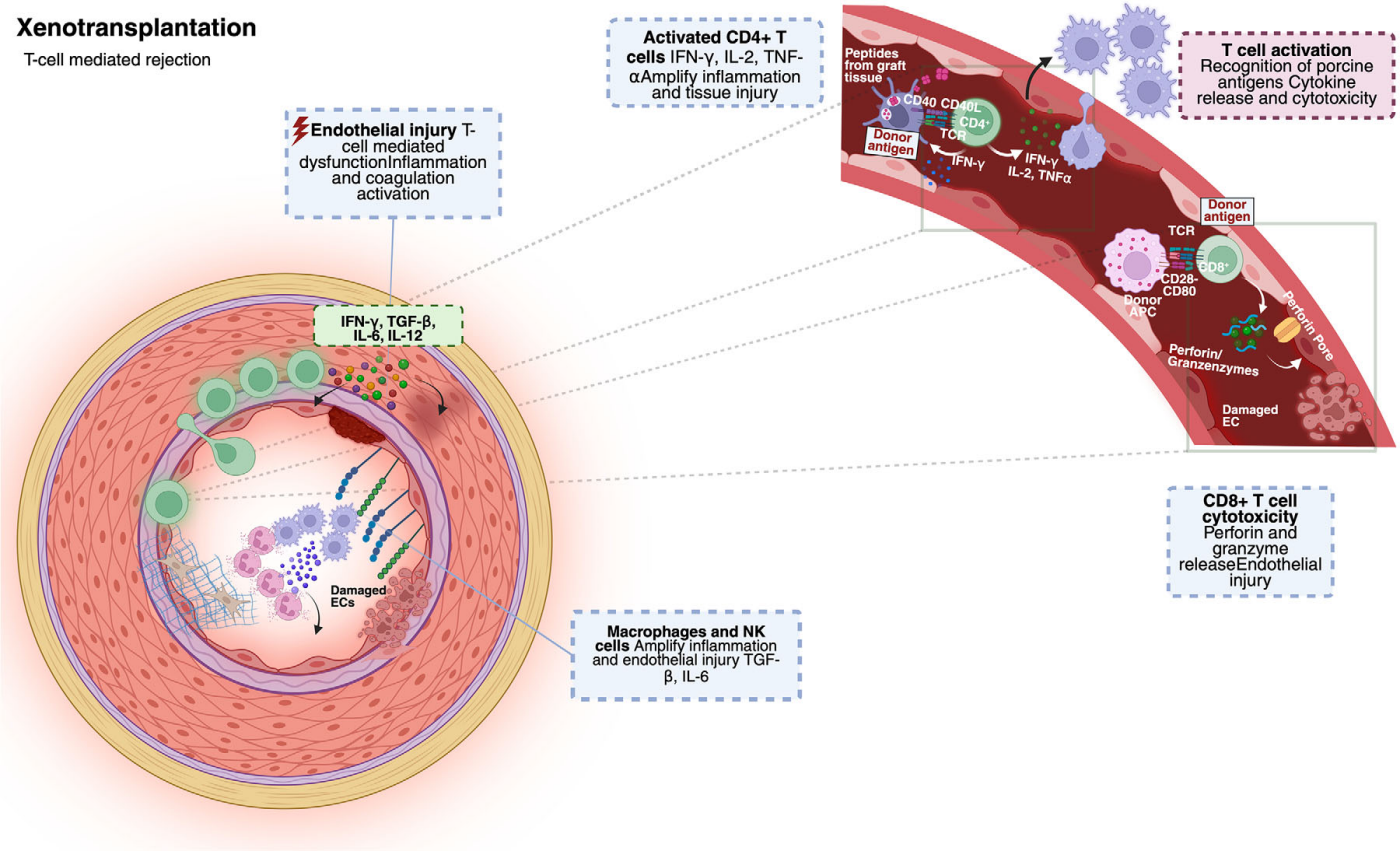
T cell‐mediated rejection in porcine lung xenotransplantation. Recognition of porcine donor antigens activates recipient CD4^+^ T helper and CD8^+^ cytotoxic T lymphocytes (CTLs). Th1 cells secrete IFN‐γ, IL‐2, and TNF‐α, amplifying the cytokine milieu and activating macrophages and NK cells, while CTLs induce endothelial apoptosis via the perforin/granzyme pathway, contributing to vascular damage. As most current lung xenografts fail early, this adaptive response is shown as a potential later barrier (proposed, dashed) rather than an established early driver. Abbreviations: CTL, cytotoxic T lymphocyte; IFN‐γ, interferon gamma; IL, interleukin; MHC, major histocompatibility complex; NK, natural killer; NKG2D, natural killer group 2D receptor; TNF‐α, tumor necrosis factor alpha.

Finally, the porcine lung differs from the human lung in its lymphocyte composition. Whereas γδ T cells constitute not more than 10% of circulating T cells in humans, they can account for up to 60% of circulating T cells in young pigs, and in porcine lung tissue they constitute 23% of CD3^+^ T cells: Comparable data for human lung tissue are scarce [22]. Porcine γδ T cells are involved in mediating stress responses and pathogen sensing, and CD8αα^+^ subsets contribute to mucosal immunity and tissue renewal. Notably, they express WC1 molecules that are absent in humans and can elicit unconventional immune reactions (Figure 1; a legend for all symbols used in Figures 1, 2, 3, 4, 5 is given in Supplementary Figure S1). Whether these species differences translate into a clinically relevant barrier in lung xenotransplantation is not yet established, but they are thought to complicate tolerance induction and may increase the risk of graft rejection.

### 2.2 Acute‐Phase Injury Mechanisms

In addition to the well‐characterized immunological pathways of rejection summarized in Figure 2, encompassing hyperacute antibody‐mediated rejection, complement activation, and adaptive cellular responses, lung xenografts are highly susceptible to non‐immune mechanisms of acute injury. These processes occur within minutes to hours after reperfusion and synergize with immune‐mediated damage, thereby accelerating graft dysfunction. Among them, ischemia–reperfusion injury (IRI) is critical because it amplifies endothelial activation, promotes the release of inflammatory mediators, and predisposes to barrier disruption. The pulmonary microcirculation, with its vast capillary surface area, is particularly vulnerable to oxidative stress and innate immune activation triggered by IRI. Closely linked to these processes, microvascular coagulopathy represents another characteristic of acute xenograft injury. Interspecies incompatibilities in coagulation and platelet regulation aggravate endothelial dysfunction and lead to diffuse thrombotic occlusion of the pulmonary vasculature [5, 23].

### 2.3 IRI and Endothelial Activation

IRI is a general consequence of organ procurement and reperfusion rather than a lung‐ or xenograft‐specific phenomenon, but the alveolar‐capillary interface is unusually vulnerable to it (Figure 3). The tissue experiences hypoxia throughout the ischemic phase, primarily promoting the accumulation of metabolic wastes such as lactate and ATP depletion, as shown in Figure 4 [24]. The hypoxic stress triggers the release of damage‐associated molecular patterns (DAMPs), such as high‐mobility group box 1 (HMGB1) and mitochondrial DNA, which in turn activates pattern recognition receptors (PRRs) including Toll‐like receptors (TLRs; TLR2, TLR4) and NOD‐like receptors (NLRs) on neutrophils and macrophages [25, 26, 27, 28, 29].

After reperfusion, oxygen is directed towards reactive oxygen species (ROS) by electron transfer chain from mitochondria to nicotinamide adenine dinucleotide phosphate (NADPH) oxidase [30]. ROS, such as hydrogen peroxide (H2O2) and the hydroxyl radical (•OH), contribute to cell damage by causing lipid peroxidation, protein carbonylation, and DNA strand breaks [31]. Furthermore, in xenografts, species incompatibilities in the CD47–SIRPα signaling axis impair the “self‐recognition” capacity of recipient macrophages. This absence of inhibitory signaling promotes excessive macrophage activation and phagocytosis, thereby amplifying early inflammation and contributing to xenograft injury during the reperfusion phase [32]. The cellular and molecular events underlying these ischemic and reperfusion‐mediated processes, from ATP depletion and mitochondrial dysfunction during cold storage to endothelial priming for the oxidative burst, are depicted in Figure 4.

### 2.4 Microvascular Coagulopathy

The lung's extensive capillary network presents a huge surface area for interactions between circulating human immune cells and the porcine endothelium. Endothelial injury at this interface is a central feature in the pathogenesis of TMA, leading to platelet activation, microthrombi formation, and subsequent organ damage [33, 34, 35]. Upon activation, neutrophils release tissue factor (TF), a potent initiator of the extrinsic coagulation cascade, which activates factor VII and promotes thrombin generation [26, 36, 37, 38]. Platelets also support thrombosis by adhering to damaged endothelial cells via P‐selectin and the GPIIb/IIIa receptor. Their activation leads to the release of ADP, thromboxane A_2_, and serotonin, which further recruits platelets and neutrophils to the site of injury. In addition, neutrophil extracellular traps (NETs) amplify the prothrombotic environment by enhancing coagulation and promoting microvascular occlusion [38]. The resulting vascular occlusion and ischemic injury augment the initial graft damage caused by IRI and accelerate graft dysfunction and failure [26, 36, 37, 39].

In pig‐to‐primate lung xenotransplantation, these mechanisms are further aggravated by molecular incompatibilities between porcine and human coagulation regulators. Porcine von Willebrand factor (vWF), for example, interacts abnormally with human GPIb, leading to fulminant platelet activation and adhesion [7]. Additionally, the expression levels and functionality of porcine thromboregulatory molecules, such as thrombomodulin, tissue factor pathway inhibitor (TFPI), and CD39, are insufficient to properly counteract the human coagulation response [14, 15].

These interspecies mismatches lead to diffuse microvascular thrombosis, particularly in the pulmonary microcirculation, and are consistently observed in failed xenografts within 1–2 weeks post‐transplantation. Despite improvements through transgenic expression of human coagulation regulators in genetically modified pigs, coagulopathy remains a major unresolved barrier in lung xenotransplantation [14, 15].

### 2.5 Complement Activation and Pathway Dysregulation

The lung is particularly sensitive to complement‐mediated injury, and despite the development of human complement regulatory protein‐transgenic pigs, complement activation remains a significant hurdle in lung xenotransplantation [7]. Complement activation is implicated in early pig‐to‐human xenograft injury via all three pathways, though direct evidence from lung‐specific models remains limited. The classical pathway is the dominant and best‐characterized one: preformed antibodies against α1,3‐Gal, N‐glycolylneuraminic acid (Neu5Gc), and Sd(a) bind C1q on the porcine endothelium, triggering C3/C5 convertase assembly and subsequently C5b‐9 deposition [7, 40]. Lectin and alternative pathways have been shown in porcine endothelial cell assays and ex vivo xenoperfusion systems, but not in lung‐specific settings to date [41]. Complement dysregulation persists even in GalTKO donors [42], and triple‐knockout grafts remain susceptible when human complement regulatory protein expression is insufficient [43].

Porcine complement regulatory proteins, such as membrane cofactor protein (MCP, CD46), decay‐accelerating factor (DAF, CD55), and membrane attack complex inhibitory protein (MAC‐IP, CD59), have substantially reduced efficacy against human complement activation due to molecular species incompatibilities, leaving the porcine endothelium poorly protected against complement‐mediated damage [40]. This vulnerability is apparent even under cell‐free conditions, consistent with the plasma‐perfusion findings noted in Section 2 [7, 15].

### 2.6 Injury Beyond the Acute Phase

While acute injury mechanisms critically determine early graft function, lung xenografts are also subject to progressive structural remodeling that extends beyond the immediate reperfusion period. These changes reflect a dynamic interplay between persistent endothelial activation, immune cell infiltration, and aberrant wound‐healing responses. As a result, histological alterations emerge that compromise pulmonary vascular integrity, promote fibrotic remodeling, and sustain chronic immune activation [7, 14, 15, 40, 44]. It should be noted that most current lung xenografts fail early from vascular barrier disruption, so direct evidence for these later remodeling processes in the lung is limited, and parts of the following are extrapolated from other organs and from general vascular biology.

#### 2.6.1 Vascular Remodeling

Although classified as a chronic process, xenograft vasculopathy in the lung begins surprisingly early, often within the first 7 to 10 days after transplantation. Several studies in pig‐to‐primate models have demonstrated that endothelial activation, intimal hyperplasia, and inflammatory cell infiltration can be detected well before overt rejection occurs. These findings suggest that vascular remodeling in the porcine lung is not merely a late‐stage phenomenon, but rather a continuous pathological process that is initiated almost immediately after engraftment and progresses in parallel with acute injury mechanisms [14, 15, 44].

Chronic xenograft vasculopathy (CXV), also known as “graft atherosclerosis”, may be initiated when the recipient's adaptive immune system recognizes porcine endothelial cells as foreign [45]. In addition, PRRs of the innate immune system detect DAMPs released by stressed or injured graft endothelial cells. These DAMPs include HMGB1, mitochondrial DNA, and heat shock proteins (HSPs).

HSPs are stress‐induced proteins that, upon release from damaged cells, act as DAMPs by activating PRRs and amplifying inflammatory signaling cascades [25, 26, 29].

Neutrophils are the primary immune cells that need to be recruited to the injury site of CXV. Selectin‐mediated adhesion, notably E‐selectin and P‐selectin, which are increased in the endothelial cell layer, facilitates the interaction between neutrophils and endothelial cells [46]. Neutrophils, on adhesion, move into the subendothelial space where they secrete proteolytic enzymes (e.g., matrix metalloproteinases (MMPs) and ROS, such as superoxide (O2), which damage the endothelial cell layer [47]. These enzymatic and oxidative by‐products damage the extracellular matrix (ECM), disrupt vascular integrity stimulate endothelial cell apoptosis, and contribute to vascular occlusion and intimal hyperplasia [47, 48].

Furthermore, chronic inflammation leads to the recruitment of macrophages, which in presence of DAMPs, including those of damaged endothelial cells polarize towards the M1 phenotype, promoting a pro‐inflammatory response through the secretion of TNF‐α, IL‐1, and IL‐6, which enhances endothelial activation [49]. In parallel, TGF‐β is secreted, primarily to activate fibroblasts, which produce collagen and contribute to fibrotic remodeling of the graft tissue, potentially compromising graft function [50]. Intimal thickening, vascular narrowing, and formation of fibroproliferative lesions which interfere with graft perfusion are characteristically associated with ischemia and ultimately leading to graft failure [26, 36, 51, 52].

#### 2.6.2 Early Fibrosis

If and if yes to what extent, classical fibrosis contributes to lung xenograft failure is still largely unknown. Present lung xenotransplantation models fail predominantly through early vascular barrier disruption, edema, hemorrhage, thrombosis, and complement and coagulation activation, rather than through mature fibrosis [7, 14, 15]. The profibrotic TGF‐β/SMAD axis, in which macrophage‐derived TGF‐β drives fibroblast activation and collagen deposition, is well described in other organs and in general tissue‐repair biology, but direct evidence in lung xenografts is lacking [50, 53, 54, 55]. It is therefore best regarded as a potential downstream process that may become relevant only if early vascular injury can be controlled and longer graft survival is achieved.

#### 2.6.3 T‐Cell Responses

As of today, the contribution of tissue‐level T‐cell responses in lung xenotransplantation remains incompletely defined. Because most current models fail early and many NHP protocols use ATG‐based lymphocyte‐depleting induction, T‐cell‐mediated injury is regarded as a potential late adaptive barrier that gains importance when prolonged graft survival can be established. The mechanisms outlined below are largely inferred from allogeneic transplantation and general immunology. Upon transplantation, xenogeneic antigens expressed on porcine endothelial cells are presented to recipient T cells via major histocompatibility complex (MHC) molecules [26, 56, 57, 58]. However, the lack of homogeneity between human and pig MHC molecules, particularly regarding class I and class II MHC expression, leads to non‐self‐awareness and activation of simultaneously CD4+ T‐helper cells and CD8+ cytotoxic T lymphocytes (CTLs) [58, 59]. Activated Th1 cells secrete IFN‐γ, TNF‐α, and IL‐2, which not only amplify the cytokine storm but also activate macrophages and neutrophils [60]. IFN‐γ induces expression of inducible nitric oxide synthase (iNOS) in both macrophages and endothelial cells, leading to increased NO production. Elevated NO levels contribute to vasodilation, endothelial dysfunction, and oxidative stress [61]. At the same time, CD8^+^ T cells recognize xenogeneic peptides presented by MHC class I molecules on porcine endothelial cells. This recognition triggers the granzyme/perforin pathway, in which perforin forms pores in endothelial cell membranes, allowing granzymes to enter the cell and induce apoptosis through caspase activation [62]. The resulting cytotoxic response promotes endothelial injury, contributing to vascular damage, microthrombus formation, and ultimately graft failure [60]. Additionally, porcine endothelial cells upregulate ligands for the activating NKG2D receptor which are recognized by human NK cells and CD8^+^ T cells, further amplifying the cytotoxic cascade depicted in Figure 5 [56, 62, 63, 64, 65].

## 3 Discussion

Despite substantial advances in donor pig genetics, survival after pig‐to‐baboon lung xenotransplantation still lags far behind cardiac and renal models. Maximum survival in the lung has reached 31 days, compared with 264 days in cardiac and 758 days in renal xenotransplantation [5, 6, 7]. This difference suggests that the lung poses specific biological challenges that are not yet adequately addressed by the genetic modifications currently in use. Multi‐transgenic donor pigs expressing human complement regulators and lacking α1,3‐Gal epitopes have reliably prevented hyperacute rejection in cardiac and renal xenotransplantation. In lung xenografts, however, the same modifications have not produced comparable results.

The published preclinical lung studies (Table 1) show a consistent pattern of mechanism‐specific gains against a background of persistent early failure. Each genetic modification has addressed a defined component of the injury cascade. Humanization of porcine vWF reduced early platelet sequestration in both ex vivo perfusion and pig‐to‐baboon transplantation, although without a clear survival benefit [66]. Expression of hTFPI together with hCD47 lowered coagulation activation, platelet activation and pulmonary edema and delayed neutrophil sequestration, while residual early platelet sequestration persisted [67]. Increasing the hCD46 gene dose and adding hCD55 produced a gene‐dose effect that improved ex vivo perfusion survival and reduced microvascular thrombosis, but complement activation measured as C3a was not reduced [68]. In the only multi‐week in vivo model, combined hEPCR and hTBM expression with anti‐inflammatory treatment extended survival to a maximum of 31 days, whereas progressive elevation of pulmonary vascular resistance, loss of vascular barrier integrity and tracheobronchial flooding remained as major injury events [7]. The first pig‐to‐human lung, transplanted into a brain‐dead recipient, maintained gas exchange over a 9‐day observation period, but developed progressive antibody‐mediated injury [69]. Taken together, platelet sequestration, coagulation activation and pulmonary edema now appear at least partially controllable, and hyperacute rejection can be prevented, whereas persistent complement activation, progressive vascular barrier failure with vascular leak and airway flooding, and, at longer survival, humoral antibody‐mediated injury remain the principal unresolved barriers.

Several lung‐specific features are likely to account for this situation. One important factor is the presence of PIMs in pigs, which have no direct equivalent in humans. Upon xenogeneic exposure, these cells mount an early innate immune response and release thromboxane and pro‐inflammatory cytokines that can damage the graft independently of adaptive immunity [20, 21]. The alveolar‐capillary interface is also highly vulnerable to IRI. In addition, lung transplantation requires the coordinated restoration of both vascular and airway function, which imposes physiological demands not seen in cardiac xenotransplantation. Structural incompatibilities between porcine and human surfactant proteins may further impair alveolar function after transplantation [19, 32]. The lung therefore presents a partly distinct set of injury mechanisms for which the current genetic and pharmacological strategies were not primarily developed. In addition, the anatomical continuity of the airways allows a localized injury to spread rapidly, for example through the spill of edema fluid, and to compromise other regions of the lung beyond the initial site. The markedly higher proportion of γδ T cells in pigs may add to this innate‐like reactivity; TRDC‐knockout pigs lacking γδ T cells have recently been generated and could be instrumental in overcoming this hurdle although they have not yet been tested in lung xenotransplantation [70].

The pharmacological studies reviewed here support this view. Selectin inhibition, DDAVP combined with anti‐GPIb strategies, and thromboxane or histamine receptor blockade each target individual components of the early rejection cascade and have shown beneficial effects in perfusion models [40, 71]. Costimulatory blockade has been highly effective in cardiac and renal xenotransplantation and anti‐CD40 is now often favored over anti‐CD154 because of the thromboembolic risk associated with the latter. In the lung, however, these approaches have not been sufficient to prevent graft failure. As summarized in Table 2, a broad range of strategies has been explored, often in complex combinations, yet survival remains limited [12, 39]. This pattern suggests that many currently used interventions target pathways shared with other organs, while key lung‐specific drivers of graft injury remain largely unaddressed.

**TABLE 2:** Genetic and pharmacological strategies targeting key injury mechanisms in porcine lung xenotransplantation.

Target mechanism	Genetic / pharmacological strategy	Evidence in lung xenotransplantation (model, key reference)
Platelet sequestration & vWF–GPIb incompatibility	Humanized pvWF (h‐pvWF); anti‐GPIb Fab; desmopressin (DDAVP); thromboxane synthase inhibition (1‐BIA); histamine receptor blockade	Human‐blood EVLP (Connolly et al., 2021; Miura et al., 2022); NHP in vivo (Burdorf et al., 2022)
Complement activation	Transgenic hCD46, hCD55, hCD59; eculizumab (C5 inhibitor)	Human‐blood EVLP – gene‐dose effect on survival (Chaban et al., 2023); brain‐dead human recipient (He et al., 2025)
Coagulation dysregulation & endothelial activation	Transgenic hEPCR, hTBM, hTFPI; alpha‐1‐antitrypsin (A1AT)	Human‐blood EVLP (Miura et al., 2022); NHP in vivo – survival up to 31 days (Burdorf et al., 2022)
Macrophage activation & phagocytosis (PIMs)	Transgenic hCD47; donor macrophage depletion (liposomal clodronate)	Human‐blood EVLP (Miura et al., 2022); pig lung xeno model (Rao et al., 2003)
Neutrophil sequestration	Selectin inhibition (rPSGL‐1, GMI‐1271); integrin inhibition (IB4)	Human‐blood EVLP – tested; no consistent reduction in neutrophil sequestration observed (Miura et al., 2022; Connolly et al., 2021)
T‐cell–mediated rejection	Anti‐CD40 mAb; tacrolimus; MMF; rATG	NHP in vivo (Burdorf et al., 2022); brain‐dead human recipient (He et al., 2025)
IRI & endothelial cytoprotection	Transgenic HO‐1 (heme oxygenase‐1)	NHP in vivo (Burdorf et al., 2022); pig lung xeno model (Martens et al., 2012)
Thromboxane and histamine‐mediated PVR elevation & vascular barrier loss	Thromboxane synthase inhibition (1‐BIA); H1/H2 histamine receptor blockade	Human blood EVLP – prevented PVR elevation and vascular barrier loss; platelet/neutrophil sequestration persisted (Burdorf et al.,2019)

Abbreviations: 1‐BIA, 1‐benzylimidazole; A1AT, alpha‐1‐antitrypsin; DDAVP, desmopressin; HO‐1, heme oxygenase‐1; h‐pvWF, humanized porcine von Willebrand factor; hCD46/55/59, complement regulatory proteins; hCD47, human CD47; hEPCR, endothelial protein C receptor; hTBM, thrombomodulin; hTFPI, tissue factor pathway inhibitor; MMF, mycophenolate mofetil; PIMs, pulmonary intravascular macrophages; rATG, rabbit anti‐thymocyte globulin.

Complement inhibition strategies, including systemic depletion with cobra venom factor, C1‐esterase inhibitor, and nafamostat, reduced complement activation in experimental models but provided only partial protection; transgenic expression of human complement regulatory proteins (hCD46, hCD55, hCD59) in GalTKO donor pigs attenuated, but did not eliminate, complement‐mediated injury, with residual lectin and alternative pathway activity persisting even in multitransgenic donors [7, 23, 40, 41, 42, 72].

A more organ‐focused strategy has emerged via ex vivo genetic modification during normothermic machine perfusion. Figueiredo and colleagues showed that lentiviral MHC silencing during ex vivo lung perfusion (EVLP) markedly reduced T‐cell alloreactivity in porcine lungs. In an allogeneic setting, transplantation of lungs with downregulated swine leukocyte antigen (SLA) expression resulted in long‐term survival without systemic immunosuppression, with some grafts functioning for up to six years [73]. These findings cannot be directly transferred to xenotransplantation, where the immunological barriers are substantially greater. The principle is nonetheless worth attention: organ‐level modification during machine perfusion may help reduce the pharmacological dosis in ways that systemic immunosuppression alone cannot achieve. This may be particularly important in lung transplantation, where excessive immunosuppression carries added risks because of the grafts continuous exposure to inhaled pathogens. Desmopressin pretreatment reduced thrombogenic responses in porcine pulmonary xenografts [74], nafamostat mesilate attenuated complement activation during ex vivo perfusion [72], and elimination of the α1,3‐Gal epitope consistently improved outcomes in human blood perfusion models [75]. In addition, meta‐analyses indicate that combining multiple genetic modifications improves lung graft function in this setting [76].

The first transplantation of a six‐gene‐edited porcine lung into a brain‐dead human recipient, reported by He and colleagues in 2025, represents an important step for the field [69, 77]. Most notably, hyperacute rejection was not observed, indicating that the current genetic backbone can prevent the most prevalent form of immune‐mediated graft failure in a human recipient. At the same time, the study design limits the conclusions that can be drawn. Only a single lung was transplanted, while the contralateral native lung remained in situ. As a result, the xenograft was not required to sustain gas exchange on its own, and its functional contribution cannot be clearly separated from that of the remaining native lung. The postoperative course also warrants caution. Severe oedema consistent with primary graft dysfunction developed within 24 h, most likely reflecting IRI. Antibody‐mediated rejection was then observed on postoperative days 3 and 6, with only partial recovery by day 9 despite intensive immunosuppression. The authors appropriately concluded that further preclinical work is needed before clinical translation can be considered. Nevertheless, this demonstrates is that hyperacute rejection did not occur in a human recipient using this six‐gene‐edited donor, which was not previously known. However, by design, it does not show whether a porcine lung can sustain adequate gas exchange in a recipient who fully depends on the graft for survival.

## 4 Conclusion

For lung xenotransplantation to advance beyond its current limitations, research will need to address the organ's specific biology more directly. Key priorities include:

1. Refinement of genetic modifications to address lung‐specific rejection mechanisms. The current multi‐transgenic backbone has successfully prevented hyperacute rejection in cardiac and renal models but remains insufficient in the lung, where pulmonary endothelial injury and capillary coagulopathy involve mechanisms not fully covered by complement regulation alone. Future pig lines should incorporate modifications targeting these lung‐specific pathways [78, 79].
2. Adaptation of immunosuppressive protocols to the lung's immunological environment. Co‐stimulation blockade has proven effective in cardiac and renal xenotransplantation, but lung xenografts show heightened susceptibility to neutrophilic infiltration and cytokine‐mediated injury, requiring more targeted immunomodulation strategies that limit early inflammatory damage without increasing infectious risk [5, 80].
3. Systematic investigation of EVLP as both a preservation and immunomodulation platform. Its dual role in reducing ischemic conditioning prior to implantation and enabling ex vivo organ‐level intervention makes it particularly suited to the physiological requirements of the lung, and its existing clinical integration provides a realistic translational pathway [81, 82, 83].
4. Development of dedicated biosafety protocols addressing the risk of aerosolized porcine endogenous retrovirus (PERV) transmission during respiratory contact, which represents a challenge without direct parallel in cardiac or renal xenotransplantation and requires surveillance strategies specifically designed for porcine lung recipients [84, 85, 86].
5. Development of tolerance‐inducing strategies adapted to the lung's continuous antigen exposure, including thymic transplantation and regulatory T‐cell delivery approaches, recognizing that these remain long‐term objectives [12].

Progress in cardiac and renal xenotransplantation has demonstrated that even major immunological obstacles can be addressed through sustained research. Whether similar advances can be achieved in the lung is still unclear. However, developments in multi‐transgenic donor pigs, lung‐tailored pharmacological strategies, and ex vivo immunomodulation provide good reason to further pursue this field.

## Generative AI Statement

“Figure 1 **| Proposed role of porcine γδ T cells (WC1^+^) in lung xenotransplantation**” created in BioRender. Roters, N. is licensed under CC BY 4.0. “Figure 2 **| Immunological rejection cascade in porcine lung xenotransplantation**” created in BioRender. Roters, N. is licensed under CC BY 4.0. “Figure 3 **| Ischemia‐reperfusion injury in porcine lung xenotransplantation**” created in BioRender. Roters, N. is licensed under CC BY 4.0. “Figure 4 **| Cold ischemia and preservation injury during porcine lung explantation**” created in BioRender. Roters, N. is licensed under CC BY 4.0. “Figure 5 **| T cell‐mediated rejection in porcine lung xenotransplantation**” created in BioRender. Roters, N. is licensed under CC BY 4.0. “Supplementary Figure S1 | Key to symbols used in Figures 1–5” created in BioRender. Roters, N. is licensed under CC BY 4.0.

## Supporting information




**Supplementary Figure S1**: Key to symbols used in Figures 1–5. Icons for cell types, antibodies, glycan antigens, complement components, and other elements used throughout the schematic illustrations. Line style indicates the level of evidence: solid = experimentally supported in lung xenografts; dashed = proposed or hypothesized.
